# Supplementary material for: DPM1 expression as a potential prognostic tumor marker in hepatocellular carcinoma
Source: PeerJ. 2020 Nov 24;8:e10307. doi: 10.7717/peerj.10307 (PMC7694566; doi:10.7717/peerj.10307)
Supplement: Supplemental Information 1 [file peerj-08-10307-s001.docx]

Supplementary Table 1

| **Similar genes for DPM1** | | | **Similar genes for DPM2** | | **Similar genes for DPM3** | |
| --- | --- | --- | --- | --- | --- | --- |
| **Gene Symbol** | | **Gene ID** | **Gene Symbol** | **Gene ID** | **Gene Symbol** | **Gene ID** |
| **UBE2V1** | ENSG00000244687.11 | | **PTGES2** | ENSG00000148334.14 | **LINC01604** | ENSG00000253716.5 |
| **MOCS3** | ENSG00000124217.4 | | **REXO4** | ENSG00000148300.11 | **CTC-425F1.4** | ENSG00000267458.1 |
| **AAR2** | ENSG00000131043.11 | | **SSNA1** | ENSG00000176101.11 | **RP11-259G18.1** | ENSG00000261575.2 |
| **RTFDC1** | ENSG00000022277.12 | | **MED22** | ENSG00000148297.15 | **RP11-178L8.7** | ENSG00000270006.2 |
| **PTPN1** | ENSG00000196396.9 | | **MAN1B1** | ENSG00000177239.14 | **SNHG19** | ENSG00000260260.1 |
| **DDX27** | ENSG00000124228.14 | | **CIZ1** | ENSG00000148337.19 | **LAMTOR4** | ENSG00000188186.10 |
| **TTI1** | ENSG00000101407.12 | | **SNRPD1** | ENSG00000167088.10 | **CTC-338M12.5** | ENSG00000250222.1 |
| **POLR3F** | ENSG00000132664.11 | | **ARPC5L** | ENSG00000136950.13 | **LAMTOR2** | ENSG00000116586.11 |
| **YTHDF1** | ENSG00000149658.17 | | **NUDT1** | ENSG00000106268.15 | **CLDND2** | ENSG00000160318.6 |
| **RNF114** | ENSG00000124226.10 | | **SURF2** | ENSG00000148291.9 | **AC005076.5** | ENSG00000224046.1 |
| **TRPC4AP** | ENSG00000100991.11 | | **CLTA** | ENSG00000122705.16 | **CTD-2659N19.2** | ENSG00000267791.1 |
| **DHX35** | ENSG00000101452.14 | | **CDCA3** | ENSG00000111665.11 | **TCEB2** | ENSG00000103363.14 |
| **ZFP64** | ENSG00000020256.19 | | **C9orf142** | ENSG00000148362.10 | **RP11-51J9.5** | ENSG00000271869.1 |
| **SLMO2** | ENSG00000101166.15 | | **NUP214** | ENSG00000126883.16 | **DNAJC27-AS1** | ENSG00000224165.5 |
| **SNRPB2** | ENSG00000125870.10 | | **PKMYT1** | ENSG00000127564.16 | **POLR2J** | ENSG00000005075.15 |
| **DIDO1** | ENSG00000101191.16 | | **ASB6** | ENSG00000148331.11 | **BOLA2B** | ENSG00000169627.7 |
| **TM9SF4** | ENSG00000101337.15 | | **MED27** | ENSG00000160563.13 | **AC004854.4** | ENSG00000234183.1 |
| **ADNP** | ENSG00000101126.15 | | **CKS2** | ENSG00000123975.4 | **RP11-670E13.6** | ENSG00000274213.1 |
| **YWHAB** | ENSG00000166913.12 | | **PSMG3** | ENSG00000157778.8 | **RN7SKP187** | ENSG00000202406.1 |
| **UBA3** | ENSG00000144744.16 | | **DPH7** | ENSG00000148399.11 | **FIS1** | ENSG00000214253.8 |
| **STK4** | ENSG00000101109.11 | | **RPL35** | ENSG00000136942.14 | **YTHDF3-AS1** | ENSG00000270673.1 |
| **OSBPL2** | ENSG00000130703.15 | | **DTYMK** | ENSG00000168393.12 | **FAM25E** | ENSG00000231122.5 |
| **CEP250** | ENSG00000126001.15 | | **WDR34** | ENSG00000119333.11 | **SNHG9** | ENSG00000255198.4 |
| **RAE1** | ENSG00000101146.12 | | **PRPF19** | ENSG00000110107.8 | **RNU1-125P** | ENSG00000252561.1 |
| **GID8** | ENSG00000101193.7 | | **POLD1** | ENSG00000062822.12 | **SNORD3A** | ENSG00000263934.4 |
| **NANP** | ENSG00000170191.4 | | **H2AFX** | ENSG00000188486.3 | **RP1-127B20.4** | ENSG00000223730.1 |
| **UBE2N** | ENSG00000177889.9 | | **CENPM** | ENSG00000100162.14 | **RP11-56P9.6** | ENSG00000254920.1 |
| **ELMO2** | ENSG00000062598.17 | | **EMG1** | ENSG00000126749.14 | **RP11-719L21.1** | ENSG00000250547.1 |
| **CSE1L** | ENSG00000124207.16 | | **ZMYND19** | ENSG00000165724.5 | **TPI1P4** | ENSG00000225455.3 |
| **XRN2** | ENSG00000088930.7 | | **SNRPA** | ENSG00000077312.8 | **RP11-323C15.1** | ENSG00000235922.1 |
| **CCDC59** | ENSG00000133773.11 | | **TRAIP** | ENSG00000183763.8 | **OR51D1** | ENSG00000197428.2 |
| **LSM14B** | ENSG00000149657.19 | | **ATIC** | ENSG00000138363.14 | **MTND1P15** | ENSG00000264168.1 |
| **TRMT6** | ENSG00000089195.14 | | **HN1** | ENSG00000189159.15 | **RP11-344N17.8** | ENSG00000230100.1 |
| **PPHLN1** | ENSG00000134283.17 | | **POLR2H** | ENSG00000163882.9 | **RP11-715L17.1** | ENSG00000233918.1 |
| **RAB22A** | ENSG00000124209.3 | | **TUBB4B** | ENSG00000188229.5 | **RN7SKP153** | ENSG00000201896.1 |
| **MAPKAPK5** | ENSG00000089022.13 | | **FANCG** | ENSG00000221829.9 | **SGOL1P2** | ENSG00000234304.2 |
| **NUP107** | ENSG00000111581.9 | | **SLC52A2** | ENSG00000185803.8 | **GTF2IP2** | ENSG00000226930.1 |
| **TAF4** | ENSG00000130699.16 | | **MYBL2** | ENSG00000101057.15 | **RNU1-92P** | ENSG00000252826.1 |
| **MGME1** | ENSG00000125871.13 | | **URM1** | ENSG00000167118.10 | **SLIT1-AS1** | ENSG00000234855.1 |
| **DNTTIP1** | ENSG00000101457.12 | | **PHF19** | ENSG00000119403.13 | **RP11-15J23.1** | ENSG00000219500.1 |
| **PHF20** | ENSG00000025293.15 | | **EIF3B** | ENSG00000106263.17 | **BUD31** | ENSG00000106245.9 |
| **ARFGEF2** | ENSG00000124198.8 | | **RECQL4** | ENSG00000160957.12 | **RP5-837I24.5** | ENSG00000234683.1 |
| **ZW10** | ENSG00000086827.8 | | **TSEN54** | ENSG00000182173.12 | **SNORD3D** | ENSG00000277947.1 |
| **RALGAPB** | ENSG00000170471.14 | | **CCDC137** | ENSG00000185298.12 | **UQCC3** | ENSG00000204922.4 |
| **PRKAG1** | ENSG00000181929.11 | | **ITPA** | ENSG00000125877.12 | **NAT16** | ENSG00000167011.8 |
| **HSPA14** | ENSG00000187522.13 | | **SNRPB** | ENSG00000125835.17 | **ZFAND2B** | ENSG00000158552.12 |
| **PDCD10** | ENSG00000114209.14 | | **TOMM5** | ENSG00000175768.12 | **MRPL24** | ENSG00000143314.12 |
| **COMMD2** | ENSG00000114744.8 | | **POP7** | ENSG00000172336.4 | **RP11-554D14.1** | ENSG00000257129.1 |
| **ZCRB1** | ENSG00000139168.7 | | **SRRT** | ENSG00000087087.18 | **RPL35P5** | ENSG00000225573.4 |
| **QRICH1** | ENSG00000198218.10 | | **SARNP** | ENSG00000205323.8 | **RP11-60C6.6** | ENSG00000257012.1 |
